# Lysine Demethylase KDM2A Promotes Proteasomal Degradation of TCF/LEF Transcription Factors in a Neddylation-Dependent Manner

**DOI:** 10.3390/cells12222620

**Published:** 2023-11-13

**Authors:** Tijana Šopin, František Liška, Tomáš Kučera, Dušan Cmarko, Tomáš Vacík

**Affiliations:** 1 Institute of Biology and Medical Genetics, First Faculty of Medicine, Charles University and General University Hospital in Prague, 128 01 Prague, Czech Republic; frantisek.liska@lf1.cuni.cz (F.L.); tijana.sopin@lf1.cuni.cz (T.Š.); dcmar@lf1.cuni.cz (D.C.); 2 Institute of Histology and Embryology, First Faculty of Medicine, Charles University and General University Hospital in Prague, 128 01 Prague, Czech Republic; tomas.kucera@lf1.cuni.cz

**Keywords:** KDM2A, canonical Wnt signaling, TCF/LEF, ubiquitination, neddylation

## Abstract

Canonical Wnt signaling is essential for a plethora of biological processes ranging from early embryogenesis to aging. Malfunctions of this crucial signaling pathway are associated with various developmental defects and diseases, including cancer. Although TCF/LEF transcription factors (TCF/LEFs) are known to be essential for this pathway, the regulation of their intracellular levels is not completely understood. Here, we show that the lysine demethylase KDM2A promotes the proteasomal destabilization of TCF/LEFs independently of its demethylase domain. We found that the KDM2A-mediated destabilization of TCF/LEFs is dependent on the KDM2A zinc finger CXXC domain. Furthermore, we identified the C-terminal region of TCF7L2 and the CXXC domain of KDM2A as the domains responsible for the interaction between the two proteins. Our study is also the first to show that endogenous TCF/LEF proteins undergo KDM2A-mediated proteasomal degradation in a neddylation-dependent manner. Here, we reveal a completely new mechanism that affects canonical Wnt signaling by regulating the levels of TCF/LEF transcription factors through their KDM2A-promoted proteasomal degradation.

## 1. Introduction

Canonical Wnt signaling, which activates its target genes through beta-catenin, is involved in a plethora of biological processes ranging from stem cell pluripotency to aging, and its malfunction frequently leads to various developmental defects and diseases, including cancer [1,2,3,4,5]. Once the pathway is activated by the binding of Wnt ligands to their receptors at the cell membrane, beta-catenin enters the nucleus, where it interacts with the TCF/LEF transcription factors TCF7L1, TCF7L2, TCF7, and LEF1 (TCF/LEFs). When in a complex with TCF/LEFs, beta-catenin attracts co-activators to activate the transcription of the TCF/LEF target genes. In the absence of Wnt ligands, beta-catenin is phosphorylated, which subsequently leads to its ubiquitination and finally to its proteasome-mediated degradation [6]. In the absence of beta-catenin in the nucleus, TCF/LEFs interact with co-repressors and act as transcriptional repressors of their target genes. The correct dosage of beta-catenin is therefore essential, and its destabilization negatively affects the transcriptional output of the pathway. In addition to the above-mentioned phosphorylation, beta-catenin levels are also negatively affected by the Setd7-mediated methylation of lysine K180 and by the KDM2A-mediated demethylation of lysine K281-354 [7,8]. TCF/LEF transcription factors undergo functionally important post-translational modifications as well [9]. For example, the acetylation of the C. elegans TCF/LEF homolog POP-1 results in its nuclear retention [10], whereas the phosphorylation of TCF/LEFs by HIPK2 or NLK results in their release from promoters, which negatively affects the transcription of their target genes [11,12,13].

The lysine demethylase KDM2A (also known as FBXL11 and JHDM1A) was originally identified as a histone lysine demethylase with specificity for the H3K36 mono- and di-methylation (H3K36me1/2) [14,15,16]. Using its CXXC domain, KDM2A binds to nonmethylated CpG islands in promoters, where it demethylates H3K36me1/2 with its N-terminal Jumonji-C demethylase domain (Jmj-C), which results in the transcriptional repression of the associated genes [17,18,19,20]. Interestingly, KDM2A has been shown to interact with and demethylate non-histone proteins such as the p65 subunit of NF-kappaB or beta-catenin, which negatively affects their activity and stability, respectively [8,21]. KDM2A interacts with various protein partners to achieve various goals through its different domains. For example, KDM2A can interact with HP1 to transcriptionally repress pericentromeric heterochromatin through the demethylase domain [22,23,24], but it can also participate in the SCF (Skp1-Cullin1-Fbox) E3 ubiquitin ligase complex to mediate the ubiquitination of various substrate proteins such as 53BP1 or PFKFB3 independently of the demethylase domain [25,26,27].

Although TCF/LEFs and their ubiquitination-dependent proteasomal degradation have been studied [28,29,30,31,32], our knowledge about the factors that regulate the stability of the TCF/LEF transcription factors, and consequently also the transcriptional output of the whole canonical Wnt pathway, is still limited. Here, we asked whether KDM2A, a member of the SCF ubiquitin ligase complex, is involved in the regulation of TCF/LEF protein levels. We show that KDM2A interacts with TCF7L2 through its CXXC zinc finger domain and promotes the proteasome-dependent degradation of TCF/LEFs in a ubiquitination-neddylation-dependent manner. Both KDM2A and canonical Wnt signaling play important roles in stem cells, and their misregulation is associated with various cancers. For example, abnormally high levels of KDM2A promote tumor cell growth in breast cancer, lung cancer, and gastric cancer [18,33,34], whereas canonical Wnt signaling is aberrantly active in cancer stem cells [35,36,37]. It is therefore essential to understand their mode of action in every possible detail.

## 2. Materials and Methods

### 2.1. Cells, Transfection, and Treatment

HEK293T cells (Sigma, St. Louis, MO, USA, 12022001) were grown in 5% CO_2_ at 37 °C in a high-glucose DMEM medium (Sigma, St. Louis, MO, USA, D6429) supplemented with 10% fetal bovine serum (ThermoFisher Scientific, Waltham, MA, USA, 10270106) and PenStrep antibiotics (Sigma, St. Louis, MO, USA, P4333-100ML). The cells were transfected with plasmids (1 μg/1 cm of the dish diameter) using Fugene6 (Promega, Madison, WI, USA, E2691) or Turbofect (ThermoFisher Scientific, Waltham, MA, USA, R0534). HEK293T cells were treated with 1 μM of BIO (6-Bromoindirubin-3′-oxime, Sigma, St. Louis, MO, USA, B1686) for 24 h to induce canonical Wnt signaling, with 10 of μM MG132 (Sigma, St. Louis, MO, USA, M7449) for 5 h to inhibit the proteasome, with 0.5 μM of MLN4924 (Sigma, St. Louis, MO, USA, 951950-33-7) for 24 h to inhibit neddylation, with 50 μM of PYR-41 (Sigma, St. Louis, MO, USA, N2915) for 4 h to inhibit ubiquitination, or with cycloheximide (CHX, 50 μg/mL, Sigma, St. Louis, MO, USA, 01810) for various time intervals to inhibit proteosynthesis.

### 2.2. Plasmids

The expression constructs were prepared by cloning the coding regions into the pCS2 plasmids. The coding regions were amplified by RT-PCR with the following primers: TCF7L2: ATGCCGCAGCTGAACGGCGGTG and CTAGTAAGCTTCCATCTGAAGAGGG; TCF7L2delN: ATGTCGAACAAAGTACCGGTGGTG and CTAGTAAGCTTCCATCTGAAGAGGG; TCF7L2delC: ATGCCGCAGCTGAACGGCGGTG and CTGGGAGGATTCCTGCTTGACTG; and beta-catenin: ATGGCTACTCAAGCTGACCTGATG and TTACAGGTCAGTATCAAACCAGGC. The plasmids were verified through sequencing. The other constructs and plasmids are described elsewhere [38].

### 2.3. RNA and q-RT-PCR

Total RNA was prepared with TRI Reagent (ThermoFisher Scientific, Waltham, MA, USA, AM9738) according to the manufacturer’s instructions and reverse transcribed with the LunaScript RT SuperMix kit (NEB, Ipswich, MA, USA, E3010S). cDNA was analyzed by quantitative PCR using a CFX96 Touch Real-Time PCR Detection System (BIO-RAD, Hercules, CA, USA), a Luna Universal qPCR Master Mix (NEB, Ipswich, MA, USA, M3003L), and the primers listed in Table 1. The results were analyzed using CFX Maestro software v2.3 (BIO-RAD, Hercules, CA, USA). The data are presented as the arithmetic means ± SD of three independent experiments. The statistical significance was determined by the student’s *t*-test as follows: * *p* < 0.05, ** *p* < 0.01, *** *p* < 0.001, n.s.—not significant.

### 2.4. Luciferase Reporter Assay

HEK293T cells were co-transfected with the pNL1.1 nanoluciferase reporter constructs, expression constructs, and control firefly constructs using Fugene6 (Promega, Madison, WI, USA, E2691). The reporter assays were performed using the Nano-Glo Dual-Luciferase Reporter Assay System (Promega, Madison, WI, USA, N1610) and the Infinite 200 luminometer (Tecan, Zürich, Switzerland). The data are presented as the arithmetic means ± SD of three independent experiments. The statistical significance was determined by the student’s *t*-test as follows: * *p* < 0.05, ** *p* < 0.01, *** *p* < 0.001, n.s.—not significant.

### 2.5. Antibodies

anti-FLAG (Sigma, St. Louis, MO, USA, F1804, Cell Signaling, Danvers, MA, USA, 14793), anti-KDM2A (Bethyl, Montgomery, TX, United States, A301-476A), anti-TCF/LEFs (Cell Signaling, Danvers, MA, USA, 9383), anti-beta-catenin (Cell Signaling, Danvers, MA, USA, 8814, 8480), anti-NEDD8 (Cell Signaling, Danvers, MA, USA, 2754), anti-Ubiquitin (Cell Signaling, Danvers, MA, USA, 43124), anti-GAPDH (Cell Signaling, Danvers, MA, USA, 2118), anti-LAMIN B1 (abcam, Cambridge, UK, ab16048), anti-rabbitCy3 (Jackson Immuno Research, West Grove, PA, USA, 711-165-152), and anti-rabbitHRP (Cell Signaling, Danvers, MA, USA, 7074), anti-mouseHRP (Sigma, St. Louis, MO, USA, GENA931-1ML) were used.

### 2.6. Proteins and Western Blot

Whole cell extracts were prepared by rotating the cell pellets for 2 h at + 4 °C in five volumes of a high-salt lysis buffer (50 mM Tris, 300 mM NaCl, 10% glycerol, 0.5% NP-40, 1× cOmplete ULTRA protease inhibitors (Sigma, St. Louis, MO, USA, 5892791001)). Proteins were resolved on 10% SDS-PAGE gels, transferred to the Immobilon-P PVDF membrane (Sigma, St. Louis, MO, USA, IPVH85R), and immunodetected with the SuperSignal West Pico PLUS Chemiluminescent Substrate (ThermoFisher Scientific, Waltham, MA, USA, 34580), and the corresponding primary and secondary antibodies are specified in 4.5. The Western blot signals were quantified using Fiji software v1.54f [39]. The TCF/LEF signals were normalized against those of the GAPDH loading control. The data are presented as the ratio between the normalized signal (the average from 3 Western blots) from the cells transfected with the empty pCS2 plasmid and those from the cells transfected with the pCS2-KDM2A plasmid. The statistical significance was determined by the student’s *t*-test as follows: * *p* < 0.05, ** *p* < 0.01, *** *p* < 0.001, n.s.—not significant.

### 2.7. Co-Immunoprecipitation and GFP-Trap

Protein extracts were prepared from the cells transfected with FLAG-tag (for Co-IP) or EGFP-tag (for GFP-trap) expression constructs, as described in 4.6., and diluted to 150 mM of NaCl and 0.1% NP-40. For Co-IP, 0.5 mg of whole cell extracts was rotated overnight at +4 °C with 2.5 μg of the anti-FLAG antibody (Sigma, St. Louis, MO, USA, F1804), and the protein-immunocomplexes were separated with Dynabeads Protein G magnetic beads (ThermoFisher Scientific, Waltham, MA, USA, 10004D). The GFP-trap experiment was carried out according to the manufacturer’s protocol (Chromotek gtak-20). Briefly, 0.5 mg of whole cell extracts from the cells overexpressing an EGFP-tagged protein was incubated with 20 μL of the EGFP-nanobody-agarose beads for 2 h at +4 °C. Tagged proteins and their protein partners were eluted by boiling the beads in the LDS sample buffer (ThermoFisher Scientific, Waltham, MA, USA, 84788) and analyzed through Western blotting.

## 3. Results

### 3.1. KDM2A Promotes Destabilization of TCF/LEF Proteins Independently of Its Demethylase Domain and in a CXXC-Domain-Dependent Manner

**Figure 1:**
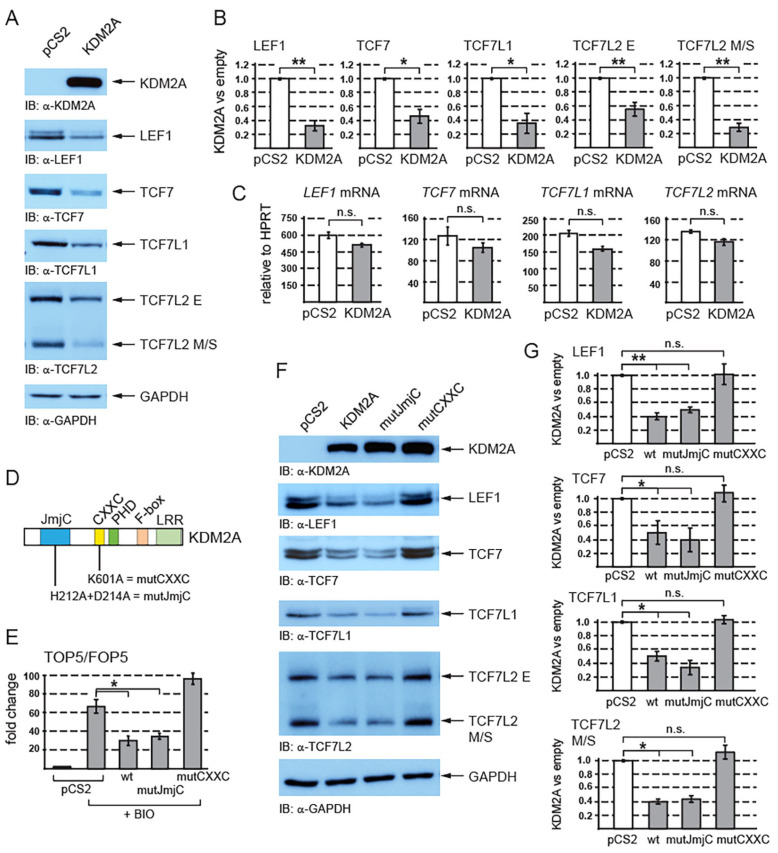
KDM2A negatively affects canonical Wnt signaling by destabilizing TCF/LEF transcription factors. (**A**) The overexpression of KDM2A in HEK293T cells was confirmed through Western blotting. Elevated levels of KDM2A result in decreased protein levels of endogenous TCF/LEF proteins. GAPDH serves as a loading control; (**B**) the overexpression of KDM2A leads to a statistically significant decrease in TCF/LEF protein levels. The Western blot signals were quantified using Fiji software v1.54f [39]. The TCF/LEF signals were normalized against those of the GAPDH loading control. The data are presented as the ratio between the normalized signal from the cells transfected with the empty pCS2 plasmid (empty) and that from the cells transfected with the pCS2-KDM2A plasmid (KDM2A); (**C**) quantitative RT-PCR confirmed that the overexpression of KDM2A does not affect the *TCF/LEF* mRNA levels when related to the HPRT1 mRNA levels. Similar results were obtained when the *TCF/LEF* mRNA levels were normalized against RPL32; (**D**) KDM2A and its domains. The JmjC demethylase domain is localized at the N-terminus (in blue), and its function is abolished by the H212A/D214A mutation (mutJmjC) [20]. The zinc finger CXXC domain (in yellow) is disrupted by the K601A mutation [20]; (**E**) the canonical Wnt signaling luciferase reporter TOP5 is activated by the pathway agonist BIO but repressed by elevated levels of both the wild-type KDM2A (wt) and the mutant KDM2A, whose demethylase domain was disrupted (mutJmjC). The mutant KDM2A protein whose CXXC domain was disrupted (mutCXXC) was not able to repress the activated reporter. The luciferase reporter activity is expressed as a fold change ratio between the normalized luciferase signal from the TOP5 transfected cells and that from the FOP5 transfected cells; (**F**) the overexpression of both the wild-type and the mutant demethylase domain defective KDM2A proteins results in lower TCF/LEF levels, whereas the mutant KDM2A protein with a disrupted CXXC domain failed to destabilize TCF/LEFs; (**G**) the overexpression of both the wild-type (wt) and the JmjC demethylase domain defective (mutJmjC) KDM2A protein results in a significant decrease in the TCF/LEF protein levels, whereas the CXXC mutant KDM2A protein (mutCXXC) does not affect the TCF/LEF protein levels. The blots were analyzed using the same approach as in (**B**). The statistical significance was determined by the student’s *t*-test as follows: * *p* < 0.05, ** *p* < 0.01 (n = 3).

We have previously shown that KDM2A negatively affects canonical Wnt signaling independently of its N-terminal JmjC demethylase domain [38]. Since KDM2A has been shown to negatively affect the stability of various proteins [25,26,27], we asked whether this lysine demethylase affects the levels of TCF/LEF transcription factors. We overexpressed KDM2A in HEK293T cells and we analyzed the TCF/LEF protein levels through Western blotting. Interestingly, our Western blot analysis showed that elevated levels of KDM2A lead to significantly reduced levels of endogenous TCF/LEF proteins (Figure 1A,B).

In the case of TCF7L2, which exists as multiple protein isoforms that all fall into two size groups, E and M/S [40], the KDM2A-associated destabilization was more prominent for the M/S isoforms. To rule out the possibility that the TCF/LEF protein levels are decreased due to KDM2A transcriptionally repressing the *TCF/LEF* promoters, we analyzed the *TCF/LEF* mRNAs by quantitative RT-PCR (q-RT-PCR). Our q-RT-PCR experiments showed that the overexpression of KDM2A does not significantly affect the *TCF/LEF* mRNA levels (Figure 1C), which supports the existence of a KDM2A-mediated mechanism that destabilizes TCF/LEF proteins.

To reanalyze the effect of KDM2A on canonical Wnt signaling, we performed luciferase reporter experiments with the luciferase reporter construct in which the luciferase gene is regulated by five TCF/LEF consensus binding sites (TOP5) or by five mutated nonfunctional TCF/LEF binding sites (FOP5) [38]. In the HEK293T cells, we overexpressed KDM2A or its mutant variants (Figure 1D), and we stimulated the canonical Wnt pathway with BIO (6-Bromoindirubin-3′-oxime). BIO inhibits the GSK3 kinase, which consequently stabilizes the nuclear beta-catenin and activates the TCF/LEF target genes [41,42]. While there was minimal luciferase activity from both TOP5 and FOP5 in the nontreated cells, the BIO-treated cells showed increased luciferase activity from TOP5, but not from FOP5. Consistently with our previously published results, the overexpression of KDM2A strongly repressed the activated TOP5 reporter independently of the KDM2A Jmj-C demethylase domain (Figure 1E).

Since KDM2A can repress the canonical Wnt signaling luciferase reporter independently of its N-terminal demethylase domain (Figure 1E), we asked first whether the KDM2A-mediated destabilization of TCF/LEFs is also independent of this domain and second whether the zinc finger CXXC domain could play some role in it. Our analyses showed that the JmjC demethylase domain is not involved in the KDM2A-mediated destabilization of TCF7L2 (Figure 1F,G). On the other hand, the mutant KDM2A protein whose CXXC domain was disrupted was not able to promote the degradation of TCF/LEFs (Figure 1F,G).

### 3.2. KDM2A Interacts with TCF/LEFs and Promotes Their Proteasomal Degradation in a Neddylation-Dependent Manner

**Figure 2:**
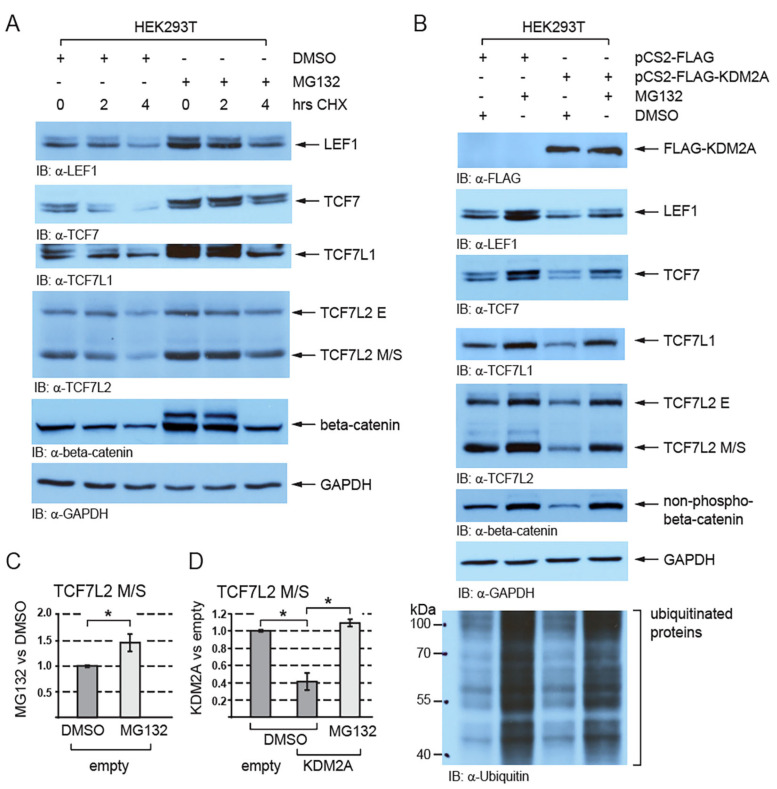
The KDM2A-mediated destabilization of TCF/LEFs is proteasome-dependent. (**A**) TCF/LEFs and beta-catenin are stabilized when the proteasome is inhibited by MG132. The cycloheximide treatment (CHX) was used to analyze the half-life of TCF/LEFs and beta-catenin; (**B**) the KDM2A-mediated degradation of TCF/LEFs and the nuclear beta-catenin (non-phospho) are prevented by blocking the function of the proteasome with MG132. Ubiquitinated proteins cannot be degraded and accumulate in the cells treated with MG132; (**C**) blocking the proteasome with MG132 stabilizes the TCF7L2 M/S isoforms and leads to their significantly higher levels. The Western blot signals were quantified using Fiji software v1.54f [39], and they were normalized against the GAPDH loading control. The data are presented as the ratio between the normalized signals from the cells treated with MG132 and those treated with DMSO; (**D**) blocking the proteasome with MG132 prevents the KDM2A-mediated degradation of TCF7LS M/S isoforms and results in their significantly higher levels in comparison to the DMSO control. Blots were quantified as in (**C**), and the data are presented as the ratio between the normalized signals from the cells transfected with the empty pCS2 plasmid (empty) and those from the cells transfected with the pCS2-KDM2A plasmid (KDM2A). The statistical significance was determined by the student’s *t*-test as follows: * *p* < 0.05 (n = 3).

Next, we asked whether the KDM2A-mediated destabilization of TCF/LEFs is proteasome-dependent. Since beta-catenin is known to undergo proteasomal degradation and to be stabilized by the proteasome inhibitor MG132 [43], it served as a positive control. Our Western blot analyses of the HEK293T cells treated with MG132 showed that TCF/LEFs are stabilized when the proteasome is inhibited (Figure 2A–C and Figure S1) and that the negative effect of KDM2A on the TCF/LEF levels is blocked by the proteasome inhibitor MG132 (Figure 2B,D and Figure S1).

**Figure 3:**
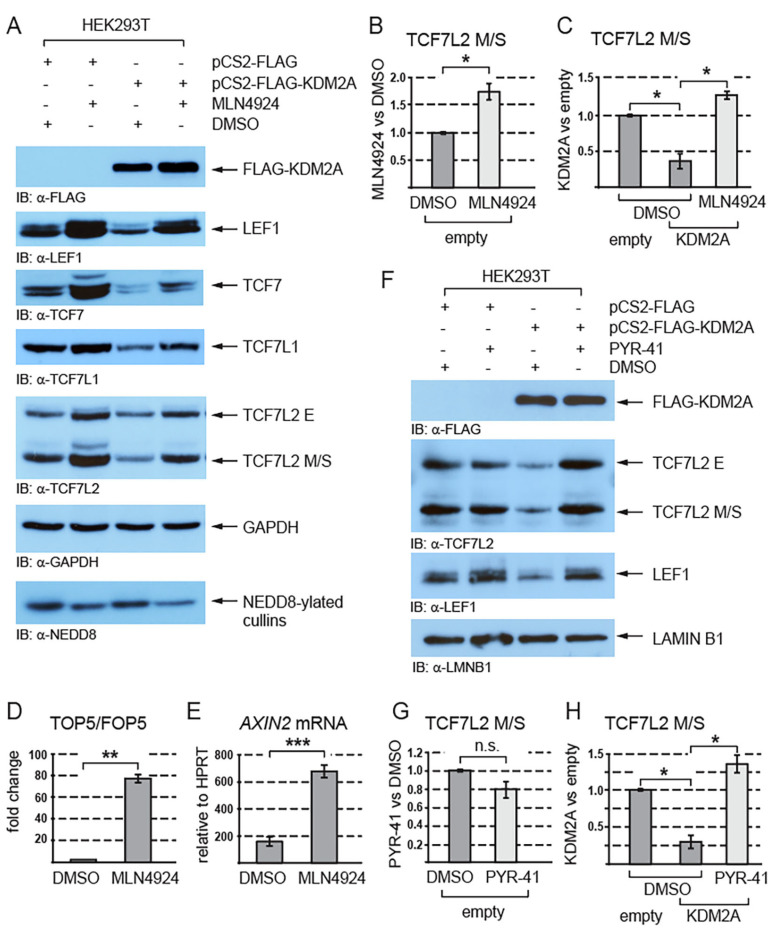
The KDM2A-mediated destabilization of TCF/LEFs is neddylation dependent. (**A**) The inhibition of neddylation with the neddylation inhibitor MLN4924 stabilizes TCF/LEFs and prevents them from being destabilized by elevated levels of KDM2A. The amount of neddylated cullins, the major NEDD8 substrate in the cell [44], is lower in the cells treated with MLN4924; (**B**) blocking neddylation with MLN4924 stabilizes the TCF7L2 M/S isoforms and leads to their significantly higher levels. The Western blot signals were quantified using Fiji software v1.54f [39], and they were normalized against the GAPDH loading control. The data are presented as the ratio between the normalized signals from the cells treated with MLN4924 and those treated with DMSO; (**C**) blocking neddylation with MLN4924 prevents the KDM2A-mediated degradation of TCF7LS M/S isoforms and results in their significantly higher levels in comparison to the DMSO control. Blots were quantified as in (**B**), and the data are presented as the ratio between the normalized signals from the cells transfected with the empty pCS2 plasmid (empty) and those from the cells transfected with the pCS2-KDM2A plasmid (KDM2A); (**D**) the TOP5 luciferase reporter is induced by the neddylation inhibitor MLN4924. The luciferase reporter activity is expressed as a fold change ratio between the normalized luciferase signal from the TOP5 transfected cells and that from the FOP5 transfected cells; (**E**) blocking neddylation with MLN4924 results in significantly higher mRNA levels of the canonical Wnt signaling target gene *AXIN2* when related to HPRT1. Similar results were obtained when the *AXIN2* mRNA levels were normalized against RPL32; (**F**) the KDM2A-mediated degradation of TCF7L2 and LEF1 is prevented when ubiquitination is blocked with PYR-41; (**G**) the TCF7L2 M/S levels are not significantly changed after treatment with PYR-41. The Western blot signals were quantified as in (**B**) using Fiji software v1.54f [39] and normalized against the LAMIN B1 loading control. The data are presented as the ratio between the normalized signals from the cells treated with PYR-41 and those treated with DMSO. (**H**) Blocking ubiquitination with PYR-41 prevents the KDM2A-promoted destabilization of the TCF7L2 M/S proteins. The signals were analyzed as in (**G**). The data are presented as the ratio between the normalized signals from the cells transfected with the empty pCS2 plasmid (empty) and those from the cells transfected with the pCS2-KDM2A plasmid (KDM2A). The statistical significance was determined by the student’s *t*-test as follows: * *p* < 0.05, ** *p* < 0.01, *** *p* < 0.001 (n = 3).

Since the proteasome degrades proteins that are post-translationally modified by ubiquitination or/and neddylation [44,45,46], we asked whether these modifications play any role in the KDM2A-mediated destabilization of TCF/LEFs. Our Western blot analyses of the cells treated with the neddylation inhibitor MLN4924 showed that inhibiting neddylation stabilizes endogenous TCF/LEFs and prevents TCF/LEFs from being destabilized by KDM2A (Figure 3A–C and Figure S2). To analyze how neddylation affects canonical Wnt signaling, we asked whether the activity of the TOP5 Wnt luciferase reporter and that of the canonical Wnt signaling target gene *AXIN2* are affected if we inhibit neddylation with the MLN4924 inhibitor. Our luciferase experiment showed that the inhibition of neddylation leads to a strong upregulation of the TOP5 reporter activity (Figure 3D). Consistently with the result of this luciferase experiment, our q-RT-PCR analysis revealed that the *AXIN2* mRNA levels are significantly higher in the cells treated with MLN4924 (Figure 3E).

Furthermore, to investigate whether the KDM2A-promoted destabilization of TCF/LEFs is ubiquitin-dependent, we analyzed the TCF7L2 and LEF1 protein levels after treatment with the ubiquitination inhibitor PYR-41. This experiment showed that the KDM2A-mediated destabilization of TCF7L2 and LEF1 is prevented when ubiquitination is inhibited by PYR-41 (Figure 3F,H and Figure S3).

**Figure 4:**
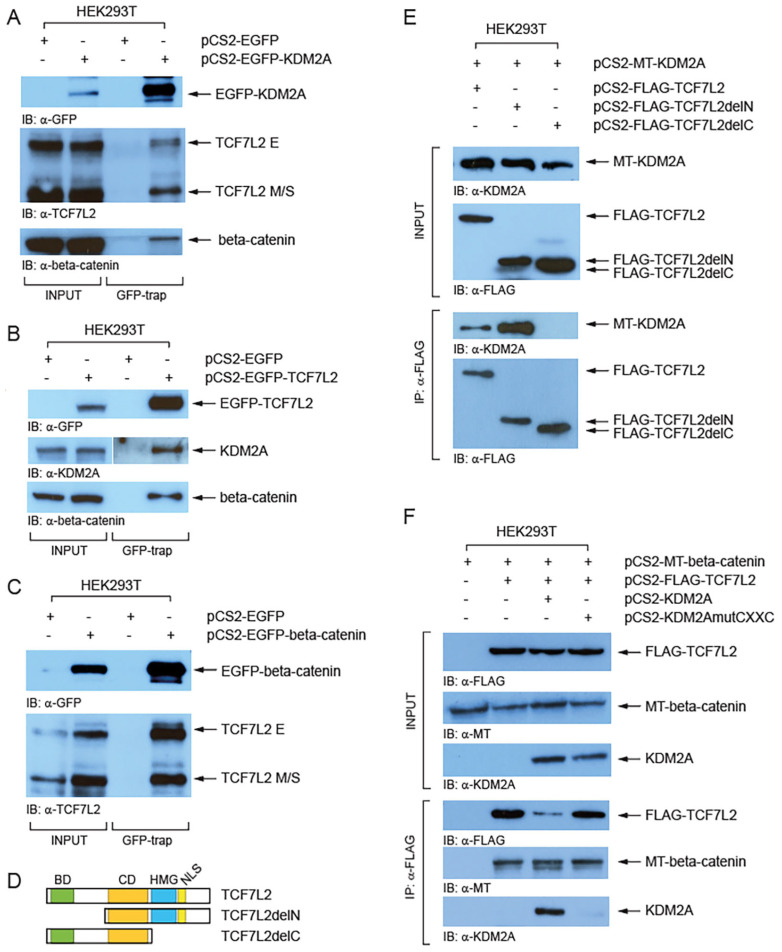
KDM2A interacts with TCF7L2 and beta-catenin. (**A**) Both TCF7L2 isoforms (E and M/S) were co-trapped with EGFP-KDM2A. The GFP-trap confirmed the interaction between beta-catenin and KDM2A; (**B**) the endogenous KDM2A and beta-catenin were co-trapped with EGFP-TCF7L2; (**C**) the endogenous TCF7L2 was co-trapped with EGFP-beta-catenin; (**D**) TCF7L2 and its domains. The beta-catenin domain (BD, in green) lies at the N-terminus. The evolutionarily conserved domain (CD, in orange) and DNA binding domain (HMG, in blue) lie in the central region. The HMG domain and the nuclear localization domain (NLS) are missing in the TCF7L2delC mutant protein; (**E**) Myc-tagged (MT) KDM2A co-immunoprecipitated with both the full-length (TCF7L2AA1-461) and the N-terminally truncated FLAG-tagged TCF7L2 (delN = TCF7L2AA162-461), but not with the C-terminally truncated FLAG-tagged TCF7L2 (delC = TCF7L2AA1-321); (**F**) KDM2A whose CXXC domain was disrupted by the K601A mutation failed to co-immunoprecipitate with FLAG-tagged TCF7L2.

To investigate whether KDM2A forms a complex with TCF/LEFs we performed a GFP-trap assay (Chromotek). Our Western blot analyses of the proteins co-trapped with EGFP-tagged KDM2A revealed that both TCF7L2 size isoform groups (E and M/S) interact with EGFP-KDM2A, although it seems more prominent for the M/S isoforms (Figure 4A). Similarly, this assay confirmed that the endogenous KDM2A forms a complex with EGFP-TCF7L2 (Figure 4B). Furthermore, our GFP-trap experiments confirmed the previously described interaction between beta-catenin and KDM2A (Figure 4A) [8] and between beta-catenin and TCF7L2 (Figure 4B,C) [47]. Next, we asked which protein domains are responsible for the interaction between KDM2A and TCF7L2. In our co-immunoprecipitation (Co-IP) experiments, myc-tagged KDM2A failed to co-immunoprecipitate with the FLAG-tagged TCF7L2 protein lacking the C-terminal region (TCF7L2AA1-321), whereas the TCF7L2 N-terminal region was not necessary for this interaction (Figure 4D,E). Moreover, our next Co-IP showed that the KDM2A protein whose CXXC domain is disrupted by the K601A mutation cannot interact with TCF7L2 (Figure 4F). Both beta-catenin and KDM2A were co-immunoprecipitated with FLAG-TCF7L2, which further confirms that the two proteins interact with TCF7L2 through different TCF7L2 domains, beta-catenin through the N-terminal beta-catenin domain, and KDM2A through the C-terminal domain (Figure 4D,E).

## 4. Discussion

Our results show that the overexpression of KDM2A leads to the destabilization of TCF/LEF transcription factors (Figure 1 and Figure 2). Since KDM2A is a CpG-island-binding transcriptional repressor [14,16], its negative effect on the TCF/LEF protein levels might be explained by KDM2A repressing the *TCF/LEF* promoters. We ruled out this possibility by analyzing the *TCF/LEF* mRNA levels, which were not significantly changed by KDM2A overexpression (Figure 1C). The negative effect of KDM2A on the TCF/LEF protein levels was therefore bound to be associated with the stability of TCF/LEFs. It has been previously shown that KDM2A negatively affects canonical Wnt signaling by destabilizing beta-catenin and that the knockdown of KDM2A stimulates the canonical Wnt signaling activity [8]. In this work, Lu et al. showed that KDM2A demethylates nuclear beta-catenin at lysines K281-354, which results in its nuclear export and proteasomal degradation. Our results indeed confirmed that the nonphosphorylated beta-catenin is negatively affected by KDM2A (Figure 2B). However, contrary to the previously published KDM2A-demethylase-domain-dependent destabilization of beta-catenin [8,48], our previous study as well as the hereby presented luciferase assay showed that KDM2A can repress the TOP5 canonical Wnt signaling reporter independently of its demethylase domain [38] (Figure 1E). Consistently with these results, our Western blot experiments showed that KDM2A promotes the degradation of TCF/LEFs independently of the demethylase domain (Figure 1F,G). Therefore, there might be a dual mode of action for KDM2A, a demethylase-dependent mode affecting beta-catenin and a demethylase-independent mode mediating the proteasomal degradation of TCF/LEFs. Moreover, our results show that the KDM2A-mediated destabilization of TCF/LEFs is dependent on the zinc finger CXXC domain (Figure 1F,G). This is consistent with our luciferase assay results, which showed that the KDM2A mutant protein whose CXXC domain was disrupted failed to repress the activated TOP5 luciferase reporter (Figure 1E) [38].

In the absence of Wnt ligands, beta-catenin is phosphorylated, ubiquitinated, and finally destroyed by the proteasome [6]. When the proteasome is blocked by MG132, beta-catenin is not degraded and accumulates in the cell [49]. TCF/LEFs have also been shown to be ubiquitinated and to undergo proteasomal degradation [28,29,30,31,32]. For example, the E3 ubiquitin ligase NARF negatively affects the stability of TCF7L2 and LEF1 by promoting their ubiquitination and consequent proteasomal degradation [29]. Our Western blot experiments confirmed that both beta-catenin and TCF/LEFs are stabilized by MG132 (Figure 2A) and further showed that the KDM2A-mediated destabilization of TCF/LEFs, as well as that of the nuclear beta-catenin, is proteasome-dependent (Figure 2B).

Neddylation, the attachment of the small ubiquitin-like protein NEDD8 to a target lysine, is a post-translational modification that marks the neddylated protein for proteasomal degradation similarly to ubiquitination [45,50]. On the other hand, the neddylation of the cullins in the SCF (Skp1-Cullin1-Fbox) E3 ubiquitin ligase complex stimulates this complex to ubiquitinate its substrates, which further leads to their proteasomal degradation [44]. Neddylation thus either directly marks proteins for proteasomal degradation or stimulates the SCF E3 ubiquitin ligase complex to ubiquitinate its substrates, which again leads to their proteasomal degradation. Interestingly, two studies have recently reported that beta-catenin is marked for proteasomal degradation by neddylation and that the inhibition of neddylation leads to increased beta-catenin levels [51,52].

Given that KDM2A is a component of the SCF E3 ubiquitin ligase complex [27], we asked whether KDM2A promotes the proteasomal degradation of TCF/LEFs in a neddylation-dependent or independent manner. Our analyses of the cells treated with the neddylation inhibitor MLN4924 showed for the first time that TCF/LEFs are stabilized when neddylation is inhibited and that the KDM2A-mediated proteasomal degradation of TCF/LEFs is neddylation-dependent (Figure 3A). Furthermore, we asked whether TCF/LEFs are degraded as a consequence of their neddylation or through ubiquitination by the SCF E3 ubiquitin ligase complex. Our degradation assay revealed that blocking ubiquitination by PYR-41 prevents the KDM2A-mediated degradation of TCF7L2 and LEF1 (Figure 3F,H and Figure S3). Taken together, our results support that KDM2A promotes the degradation of TCF7L2 and LEF1 through the SCF E3 ubiquitin ligase complex in a neddylation-dependent manner.

Next, we asked whether KDM2A and TCF/LEFs can form a complex. Our GFP-trap experiments confirmed the known interaction between beta-catenin and TCF7L2 and between beta-catenin and KDM2A and showed that KDM2A can interact with TCF7L2 (Figure 4). Our Co-IP results further showed that KDM2A interacts with TCF7L2 through its CXXC zinc finger domain (Figure 4F). This was a bit surprising since the KDM2A CXXC zinc finger domain has been described to function as a DNA-binding domain [20]. However, Yang et al. recently showed that the CXXC domain of KDM2B, another lysine demethylase and a highly similar paralog of KDM2A (93.6% amino acid similarity in their CXXC domains) [14], is indispensable for the interaction with ERRalpha [53]. The KDM2A CXXC domain thus seems to serve a double purpose—as a DNA-binding domain, through which KDM2A interacts with CpG islands, and as a protein–protein interaction domain, through which KDM2A can interact with TCF/LEFs.

## 5. Conclusions

Since canonical Wnt signaling is frequently misregulated in various cancers, including cancer stem cells [35,36,37], it is essential to understand every possible detail of its regulation. In this study, we describe a completely novel mechanism that regulates TCF/LEF protein levels and consequently, also the canonical Wnt pathway activity through their KDM2A-mediated proteasomal degradation in a neddylation-dependent manner. In the future, it will be important to investigate which TCF/LEF regions and, more specifically, which amino acid residues are responsible for the KDM2A-mediated destabilization and whether these amino acid residues are mutated in certain types of cancer. Moreover, since abnormal neddylation is known to be associated with various cancers [54,55], it would be interesting to investigate whether TCF/LEF protein levels are also inversely correlated with high neddylation in these pathological states.

## Figures and Tables

**Table 1:** The primers used in the quantitative RT-PCR assays.

Gene	Primer	Sequence (5′ to 3′)
*HPRT1*	forward	TCTTTGCTGACCTGCTGGATTAC
	reverse	GTCTGCATTGTTTTGCCAGTGTC
*RPL32*	forward	CTCAGACCCCTTGTGAAGCC
	reverse	TTGCTTCCATAACCAATGTTGG
*LEF1*	forward	GGTGAACGAGTCTGAAATCATCC
	reverse	TGTTCTCTGGCCTTGTCGTG
*TCF7*	forward	ACGAACATTTCAACAGCCCAC
	reverse	TCAGGGAGTAGAAGCCAGAGAGG
*TCF7L1*	forward	TTCCTGATGATCCCGGACC
	reverse	GAGATGGTGACCTCGTGTCCTT
*TCF7L2*	forward	GACAAGCCCTCAAGGATGCC
	reverse	CGTCGGCTGGTAAGTGTGG
*AXIN2*	forward	CTGACGGATGATTCCATGTCC
	reverse	GGGAAATGAGGTAGAGACACTTGG

## Data Availability

Data are contained within the article and Supplementary Materials.

## Supplementary Materials

The following supporting information can be downloaded at: https://www.mdpi.com/article/10.3390/cells12222620/s1.
