# Supplementary figures and images for: Lysine Demethylase KDM2A Promotes Proteasomal Degradation of TCF/LEF Transcription Factors in a Neddylation-Dependent Manner
Source: Cells. 2023 Nov 13;12(22):2620. doi: 10.3390/cells12222620 (PMC10670284; doi:10.3390/cells12222620)

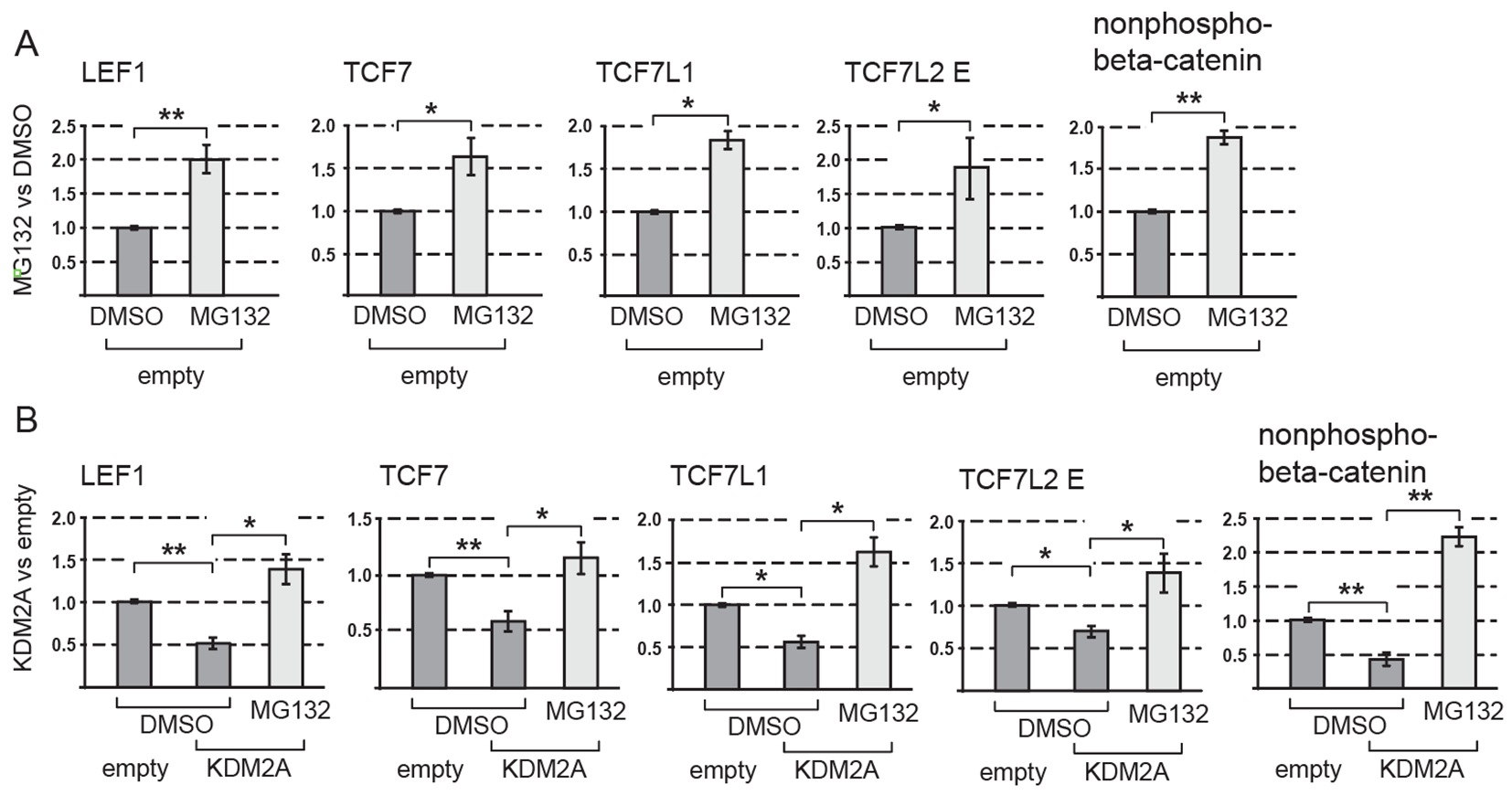

Supplement: Supplementary file 1 [file cells-12-02620-s001.zip › FigS1 resubmission.tif]

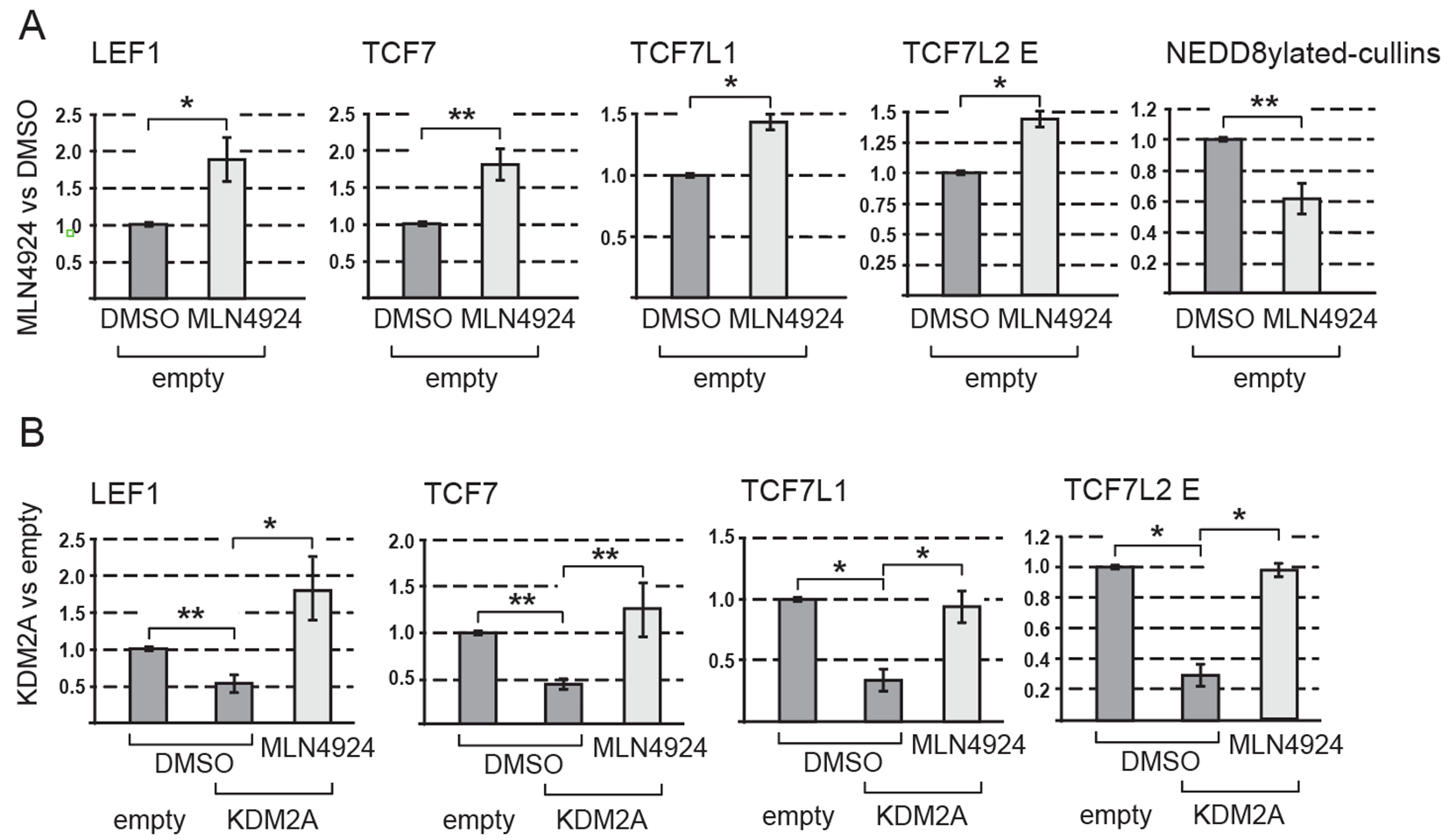

Supplement: Supplementary file 1 [file cells-12-02620-s001.zip › FigS2 resubmission.tif]

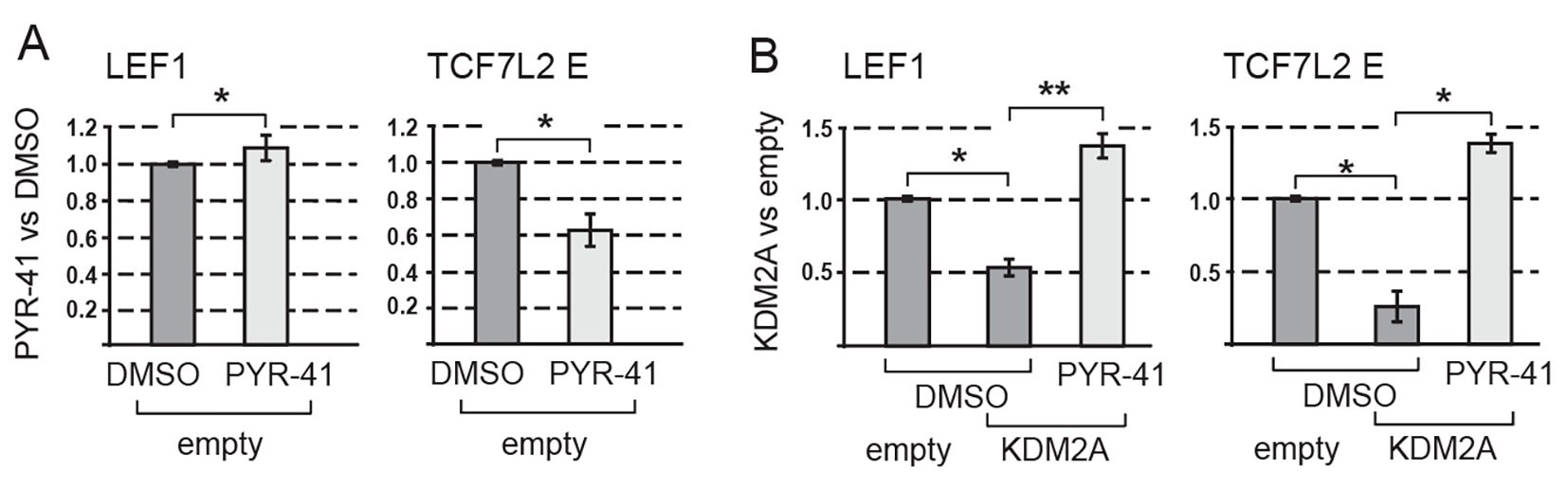

Supplement: Supplementary file 1 [file cells-12-02620-s001.zip › FigS3 resubmission.tif]
